# Characterization of a cuproptosis-related signature to evaluate immune features and predict prognosis in colorectal cancer

**DOI:** 10.3389/fonc.2023.1083956

**Published:** 2023-06-13

**Authors:** Lei Li, Fengyuan Sun, Fanyang Kong, Yongpu Feng, Yingxiao Song, Yiqi Du, Feng Liu, Xiangyu Kong

**Affiliations:** ^1^ Digestive Endoscopy Center, Shanghai Tenth People’s Hospital, Shanghai, China; ^2^ Department of Gastroenterology, Shanghai Tenth People’s Hospital, Tongji University School of Medicine, Shanghai, China; ^3^ Department of Gastroenterology, Changhai Hospital, Naval Medical University, Shanghai, China; ^4^ Clinical Research Unit, Changhai Hospital, Naval Medical University, Shanghai, China

**Keywords:** cuproptosis, prognosis, immune infiltration, elesclomol, colorectal cancer

## Abstract

**Purpose:**

Cuproptosis is a newly discovered type of cell death. Little is known about the roles that cuproptosis related genes (CRGs) play in colorectal cancer (CRC). The aim of this study is to evaluate the prognostic value of CRGs and their relationship with tumor immune microenvironment.

**Methods:**

TCGA-COAD dataset was used as the training cohort. Pearson correlation was employed to identify CRGs and paired tumor-normal samples were used to identify those CRGs with differential expression pattern. A risk score signature was constructed using LASSO regression and multivariate Cox stepwise regression methods. Two GEO datasets were used as validation cohorts for confirming predictive power and clinical significance of this model. Expression patterns of seven CRGs were evaluated in COAD tissues. *In vitro* experiments were conducted to validate the expression of the CRGs during cuproptosis.

**Results:**

A total of 771 differentially expressed CRGs were identified in the training cohort. A predictive model termed riskScore was constructed consisting of 7 CRGs and two clinical parameters (age and stage). Survival analysis suggested that patients with higher riskScore showed shorter OS than those with lower (*P<*0.0001). ROC analysis revealed that AUC values of cases in the training cohort for 1-, 2-, and 3-year survival were 0.82, 0.80, 0.86 respectively, indicating its good predictive efficacy. Correlations with clinical features showed that higher riskScore was significantly associated with advanced TNM stages, which were further confirmed in two validation cohorts. Single sample gene set enrichment analysis (ssGSEA) showed that high-risk group presented with an immune-cold phenotype. Consistently, ESTIMATE algorithm analysis showed lower immune scores in riskScore-high group. Expressions of key molecules in riskScore model are strongly associated with TME infiltrating cells and immune checkpoint molecules. Patients with a lower riskScore exhibited a higher complete remission rate in CRCs. Finally, seven CRGs involved in riskScore were significantly altered between cancerous and paracancerous normal tissues. Elesclomol, a potent copper ionophore, significantly altered expressions of seven CRGs in CRCs, indicating their relationship with cuproptosis.

**Conclusions:**

The cuproptosis-related gene signature could serve as a potential prognostic predictor for colorectal cancer patients and may offer novel insights into clinical cancer therapeutics.

## Introduction

Colorectal cancer (CRC) is the third most common cancer and the second leading cause of cancer-related deaths worldwide, with more than 1.85 million cases and 850,000 deaths annually occurred (1). Among people diagnosed with CRC, 20% have metastatic CRC, and 40% patients with localized disease will have a relapsing metastasis after curative surgical resection. The 5-year survival rate for those diagnosed with metastatic CRC is less than 20% (1, 2). To improve the prognosis of patients with CRC, there is an urgent need to develop more efficient prognostic models and targeted therapy against CRC.

Regulated cell death (RCD) is generally regulated by signaling molecules and has unique biochemical, morphological, and immunological characteristics (3). Different forms of RCD, including apoptosis, necroptosis, autophagy, ferroptosis, pyroptosis, alkaliptosis, and etc., have been identified to be involved in diverse pathological processes, including tumorigenesis (4). Certain RCD forms are regarded as targets of almost all treatment strategies. Resistance to these RCDs are common causes for failure of cancer treatment. Different forms of RCDs can be alternative therapeutics to each other to conquer treatment resistance (5). Therefore, finding new forms of RCD will bring novel therapeutics for refractory cancer cases.

Copper is an essential cofactor for all organisms, and yet it becomes toxic if concentrations exceed a threshold maintained by evolutionally conserved mechanisms (6). Accumulating evidence suggests that organic chelators of copper, e.g., elesclomol, can induce cellular copper overload and restrain malignant behaviors across various cancer types, including CRC (7). However, detained mechanisms underlying copper-related anticancer effects remain poorly understood. Different publications raised contradictory opinions, including either induction of ferroptosis (7), autophagy (8), apoptosis (9), or inhibition of the aerobic glycolysis pathway (10). Recently, Tsvetkov et al. established that copper induced death, namely cuproptosis, was a totally distinct RCD form from previous identified ones, e.g., apoptosis, ferroptosis, and necroptosis. They also showed that cuproptosis occurs by means of direct binding of copper to lipoylated components of the tricarboxylic acid cycle. Ten pivotal genes were identified involved in cuproptosis through whole-genome CRISPR-Cas9 selection screen, including seven genes (FDX1, LIAS, LIPT1, DLD, DLAT, PDHA1 and PDHB) conferred resistance to cuproptosis, while three genes (MTF1, GLS and CDKN2A) sensitized the cells to cuproptosis. Mounting evidence showed that those cuproptosis associated molecules, including noncoding genes, exhibited strong association with prognosis and immune infiltration levels.

**Figure 1:**
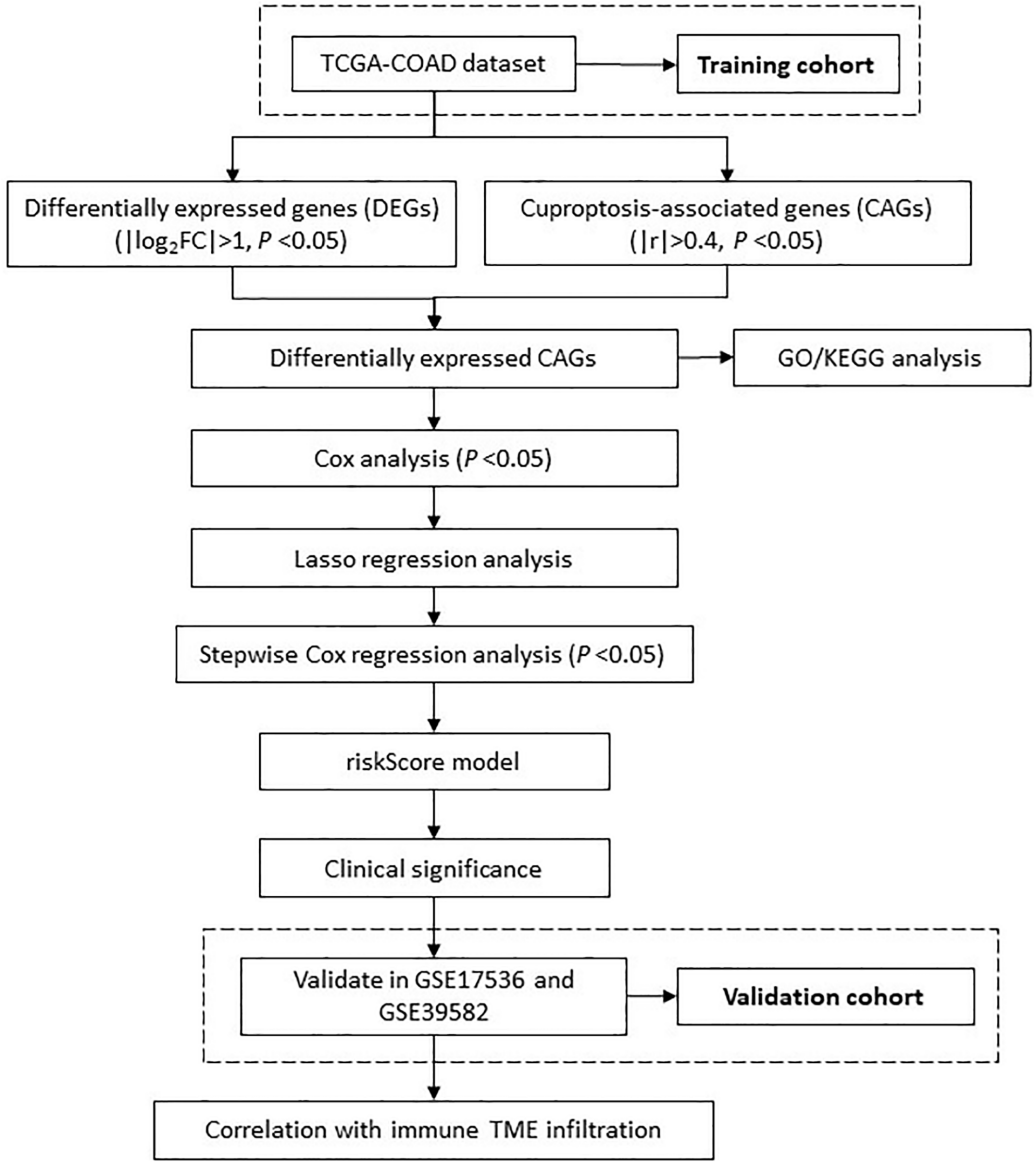
The flowchart shows the overall analytical process of this study.

In current study, we defined a list of cuproptosis associated genes (CRGs) as candidate molecules, and further developed a predictive model through LASSO regression and multivariate Cox stepwise regression in TCGA dataset. We further evaluated its associations with a list of clinical parameters, e.g., TNM stages, overall survival, treatment response, immune infiltration levels, and etc., to test its predictive efficacy and relationship with immune microenvironment features. An overview of the research design was presented in **Figure 1**.

## Material and methods

### Data collection and preprocessing

Gene expression data and clinical feature of colon cancer samples were collected from publicly available datasets of the NCBI GEO database and TCGA. A total of three colon cancer expression profile cohorts were included in our study, including GSE17536, GSE39582 and TCGA-COAD cohorts. We downloaded the normalized matrix files of each GEO cohort for further analyses (https://www.ncbi.nlm.nih.gov/geo/). For RNA sequencing data from TCGA, we downloaded the read counts of gene expression from the Xena Genomic Data Commons (http://xena.ucsc.edu/), including 471 tumor and 41 normal samples. Study participants with incomplete clinical information were excluded for further survival analysis.

### Identification of differentially expressed genes

Differential expression analysis was carried out using R package DESeq2 (11) on the 41 paired samples. Genes that showed significantly differential expression (*P*<0.05 and |log_2_ fold‐change| >1) between paired tumor and normal samples were selected for downstream analysis.

### Identification of cuproptosis-related genes

We then assessed the correlation of DEGs with 10 key cuproptosis regulators (CDKN2A, DLAT, DLD, FDX1, GLS, LIAS, LIPT1, MTF1, PDHA1 and PDHB) by Pearson’s correlation analysis. In order to identify cuproptosis-related genes, absolute Pearson’s correlation coefficients higher than 0.4 and *P* values less than 0.05 were required.

### Functional annotation and gene set enrichment analysis

To explore potential biological processes related to the obtained cuproptosis-related DEGs, we performed gene ontology (GO) and KEGG enrichment analysis using the ClusterProfiler R package (12). The GO enrichment analysis was conducted based on three aspects including biological process (BP), molecular functions (MF) and cellular components (CC). We also identified the activated or inactivated biological pathways among patients with low- and high-riskScore by running the gene set enrichment analysis (GSEA) of the adjusted expression data for all transcripts. The used gene sets were downloaded from MSigDB database, and the “c5.go.bp.v7.5.1.symbols” gene sets were used to quantify the activity of biological pathways, which was represented by the enrichment score.

### Survival analysis

Survival analysis was performed using univariate and multivariate Cox regression hazard analysis and survival curves derived from Kaplan–Meier survival analysis by using the packages survival and survminer. The receiver operating characteristic curve (ROC) were performed with the timeROC packages.

### CRG-related risk signature construction and validation

TCGA-COAD dataset was set as the training cohort to screen for those survival-related genes in COAD. Univariate Cox regression analysis was performed to screen out OS-related DEGs. LASSO regression analysis was further applied to refine DEGs, and multivariate Cox regression hazard analysis (backward stepwise) was eventually used to establish a predictive model, performance of which was ultimately validated in two independent GEO datasets.

Risk score was computed with the following equation:


$$riskScore\;=\sum_{i=1}^{n}\left(Coef_{i}\times x_{i}\right)$$

### Establishment and validation of a nomogram scoring system

We later created a hybrid nomogram using the regplot R package that incorporates the mRNA signature and clinicopathological features of COAD patients to predict their OS (1-, 3-, and 5-year). For determining the predictive power of a nomogram, calibration curves and consistency indices (C-index) were used.

### Evaluation of intratumoral immune cell infiltration

ssGSEA was used to quantify the abundance of each TME cell infiltration based on the gene sets obtained from the study of Charoentong (13). To control the bias resulted by the tumor purity, we adjusted the enrichment scores of each TME cell subtype by calculating the tumor purity using ESTIMATE algorithm. The adjusted enrichment scores calculated by ssGSEA analyses were used to represent the abundance of each TME infiltration cell.

### Mutation analysis

Somatic mutation data of COAD from whole exome/genome sequencing (WXS/WGS) were downloaded from the GDC TCGA-COAD project on the UCSC Xena server. Oncoplot was drawn according to the descending order of mutations using the R package “ maftools” (14).

### Cell culture

HCT116 and SW480 were cultured in Dulbecco’s modified Eagle medium which was supplemented with 100 U*mL-1 penicillin and streptomycin as well as 10% fetal bovine serum in a humidified atmosphere of 5% CO_2_ at 37°C.

### Reagents and drug treatment *in vitro*

Elesclomol was purchased from Master of Bioactive Molecules (MCE). When cells were adherent and had morphologically spread, Colon cancer cell lines (HCT116 and SW480) were treated with 2μM copper chloride and/or 40nM elesclomol for 24 hours, respectively. Cells were harvested after treatment and RNA was collected *via* the following extraction method.

### RNA extraction and quantitative real-time polymerase chain reaction

Total cellular RNA was extracted using a total RNA extraction kit (220010, Shanghai Feijie) according to standard protocol. The RNA was used to synthesize complementary DNA (cDNA) with a cDNA Synthesis SuperMix (RR036A, TaKaRa). The cDNA was used as a template and the seven cuproptosis related genes (GRGs) expression was quantified with the Roche LightCycler 480 using TB Green Premix Ex Taq II (RR820A, TaKaRa). GAPDH was used as an endogenous control. Primers were synthesized by Sangon Biotech (Sangon, Shanghai). The primer sequences are shown in **Table 1**.

**Table 1:** PCR primer sequences of target genes.

Primer	Sequence (5' to 3')
DPP7-F	GGACCACTTCAACTTCGAGC
DPP7-R	GCCCTCGTTCCCAGTGTAG
GPRASP1-F	AGGAGGAGACCAATATGGGGT
GPRASP1-R	GGACCTAGACATGGTATTAGCCT
UNC5C-F	TGGGACTGGGATACTTGCTG
UNC5C-R	ACAGTACAGGTTCACAGGCTTAT
CDR2L-F	TGGGCTGACGGAGACCATT
CDR2L-R	TGTAGGCGGAAAGCATCCTTG
RAB3B-F	CCGCTATGCTGATGACACGTT
RAB3B-R	ACGGTAGACTGTCTTCACCTTG
PCDH9-F	CTGCTCTGATTGCCTGTTTAAGG
PCDH9-R	ACCAGTCTGTAGACAAGGCTG
SLC18A2-F	CGGAAGCTCATCCTGTTCATC
SLC18A2-R	CCTGGCCGTCTGGATTTCTG
GAPDH-F	GGAGCGAGATCCCTCCAAAAT
GAPDH-R	GGCTGTTGTCATACTTCTCATGG

### Statistical analyses

The data were analyzed with R software version 4.2.0. For comparisons, data conforming to normal and nonnormal distributions were assessed using the unpaired/paired Student’s t-test and the Wilcoxon test, respectively. The difference significance test for three or more groups was performed using One-way ANOVA and Kruskal-Wallis tests. All statistical *P* value were two-side, with *P*< 0.05 as statistically significance.

## Results

### Identification of CRGs in TCGA-COAD cohort

**Figure 2:**
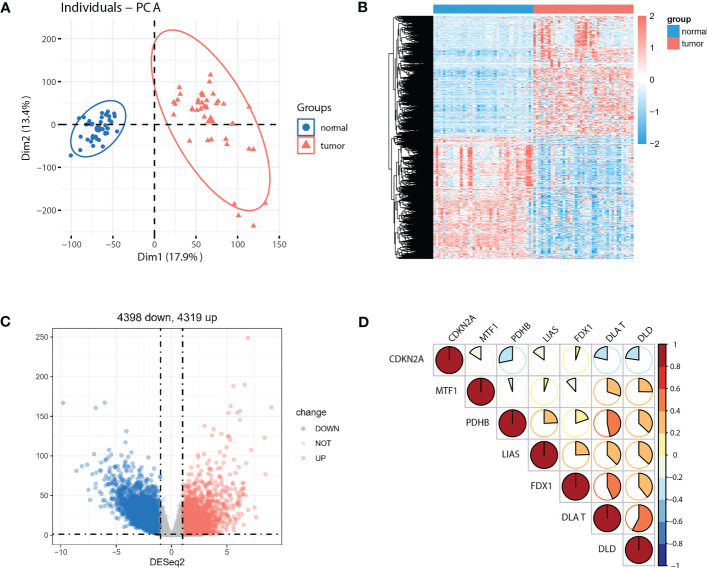
Identification of DEGs between paired normal and tumor cases in TCGA-COAD cohort. **(A)** PCA of the global transcriptome in tumor (red) and paired normal (blue) cases. A heatmap **(B)** and volcano plot **(C)** of significantly (*P*<0.05) upregulated (log2 fold-change >1, red) and downregulated (log2 fold-change<−1, blue) genes in tumor vs. normal cases. **(D)** Seven out of ten reported CRGs were differentially expressed between tumor and paired normal cases (*P*<0.05).

As the sample size of normal cases is relatively small (41 out of 512 cases), we employed paired tumor vs. normal samples to improve detection rate for differentially expressed genes (DEGs). Principal component analysis (PCA) of the full transcriptomes identified differential grouping between two cohorts (**Figure 2A**). Differential expression analysis identified 4319 significantly upregulated and 4398 significantly downregulated transcripts in CRC tissues compared with paired normal tissues (**Figures 2B, C**) at a *P*<0.05 and a |log2 fold-change| >1. Of note, seven out of ten pivotal CRGs, which were identified in *Science* article (6), showed significantly altered expression patterns, with CDKN2A exhibited higher and DLAT, DLD, FDX1, LIAS, MTF1, PDHB exhibited lower expression in tumor tissues (**Supplementary Figure 1A**, *P<*0.05). Correlations across these seven molecules in CRCs were shown in **Figure 2D**. Survival analysis showed that five out of seven molecules were significantly associated with overall survival (*P<*0.05, **Supplementary Figure 1B**).

**Figure 3:**
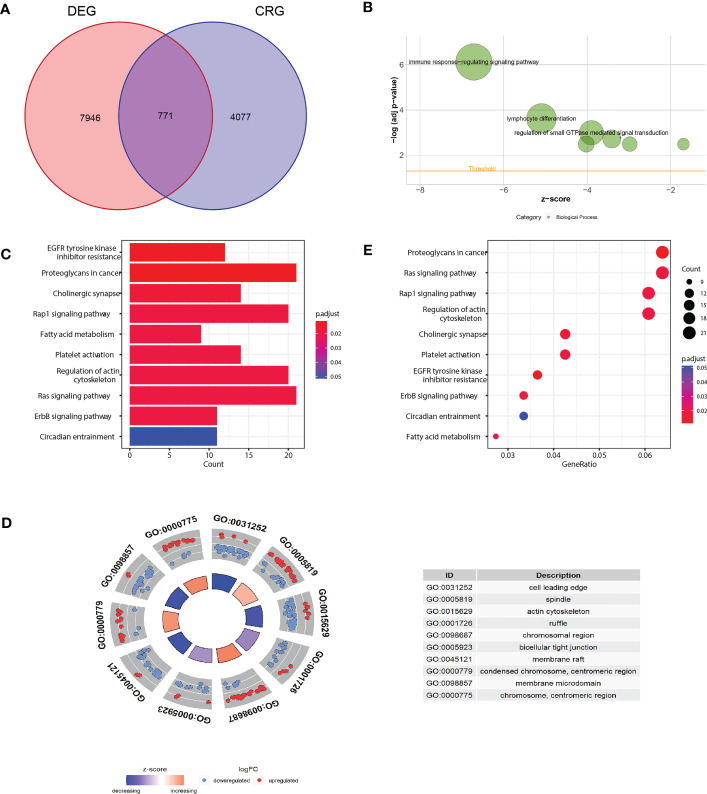
Functional annotation of differentially expressed CRGs. **(A)** Venn diagram analysis demonstrated genes appeared both in DEGs and CRGs. **(B-D)** Gene Ontology functional enrichment analyses for differentially expressed genes. **(B)** Biological processes enrichment. **(C)** Cellular component enrichment. **(D)** Molecular function enrichment. **(E)** KEGG pathway enrichment analyses for differentially expressed CRGs. All enriched pathways were significant. The color depth represented enriched adjusted *P* value.

As the concept of Cuproptosis has just recently been proposed (6) and no database are available to download the full picture of CRGs, here we used coexpression strategy (15) to define mRNAs with 7 reported CRGs absolute coefficients values >0.4 and P values<0.05 as the standard of CRGs. A total of 7946 genes were identified CRGs. Further Venn diagram showed 771 overlapping genes in DEGs and CRGs (**Figure 3A**), which we select as candidates for constructing a prognosis predictive signature. Functional annotations of GO enrichment indicated these genes were significantly associated with TME immune related biological processes such as B cell receptor signaling pathway, humoral immune response, production of molecular mediator of immune response, positive regulation of B cell activation, leukocyte migration, suggesting these CRGs could be significantly correlated with TME immune cell infiltration (**Figures 3B-D**). Consistently, KEGG pathway analysis also demonstrated that these genes were correlated with immune related signaling pathways (**Figure 3E**).

### Establishment of risk model for prognosis prediction based on CRGs

**Figure 4:**
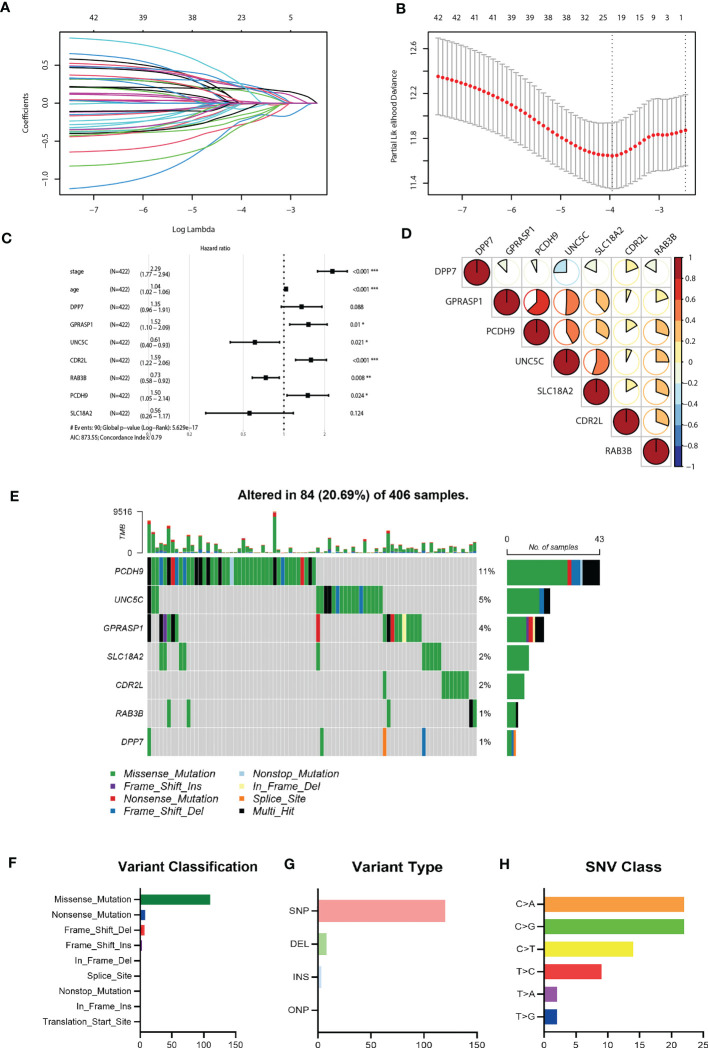
Construction of the riskScore signature and mutation analysis of seven riskScore-associated molecules. **(A)** Least absolute shrinkage and selection operator (LASSO) coefficient profiles of the 45 key molecules. **(B)** Tuning parameter selection by tenfold cross-validation in the LASSO model. The partial likelihood deviance was plotted against log(Lambda/λ), and λ was the tuning parameter. The partial likelihood deviance values were shown and error bars represented s.e. The dotted vertical lines showing the optimal values through minimum criteria and 1 -s.e. criteria. **(C)** Multivariate Cox regression analysis of seven CRGs and two clinical parameters **(D)** Correlation between seven riskScore-associated molecules. Blue, negative correlation; Red, positive correlation. **(E)** The mutation landscape of key molecules in 406 samples of TCGA-COAD cohort. **(F-H)** the CNV and mutation frequency and classification of seven prognosis-related CRGs in Colorectal cancer. *P<0.05, **P<0.01, ***P<0.001.

Considering the markedly differential expression patterns of these CRGs, we set TCGA-COAD dataset as the training cohort to screen for those survival-related genes (n = 44, *P<*0.05 both in log-rank test and in univariate Cox regression analysis). We used LASSO Cox regression to distinguish those most informative prognostic mRNA biomarkers for prognosis. Regression coefficients of the 44 DEGs were evaluated (**Figure 4A**; **Supplementary Table 1**). It was finally verified through cross-validation that 20 (**Supplementary Table 1**) DEGs could achieve a better effect in the model (**Figure 4B**). Eventually, multivariate Cox stepwise regression method was used to establish several multivariate regression models. A risk model consisting of 7 DEGs (DPP7, GPRASP1, UNC5C, CDR2L, RAB3B, PCDH9, SLC18A2), as well as two clinical parameters (age and stage), was at last identified (**Figure 4C**). DPP7, CDR2L exhibited higher and UNC5C, RAB3B, SLC18A2, GPRASP1, PCDH9 exhibited lower expression in tumor tissues (**Supplementary Figure 2A**, *P<*0.05). Survival analysis showed that UNC5C, RAB3B, SLC18A2 were low-risk genes, while DPP7, GPRASP1, CDR2L, PCDH9, were high-risk genes (*P<*0.05, **Supplementary Figure 2B**). The correlation analysis was performed to investigate the similarities among seven key molecules, and the results are visually displayed in **Figure 4D**.

Somatic mutation profiles of 7 key genes for 406 CRC patients were retrieved from the TCGA dataset. The waterfall plot was used to present the mutation data for each gene in every sample (**Figure 4E**), Further, mutations were grouped based on various categories. In the grouping, missense mutation was the most common (**Figure 4F**), while single nucleotide polymorphisms (SNP) is more common than other kinds of mutations (**Figure 4G**). Regarding single nucleotide variants, C>A and C>G are two most common kinds (**Figure 4H**).

### Evaluation of the predictive efficacy of the riskScore for prognosis in TCGA-COAD

**Figure 5:**
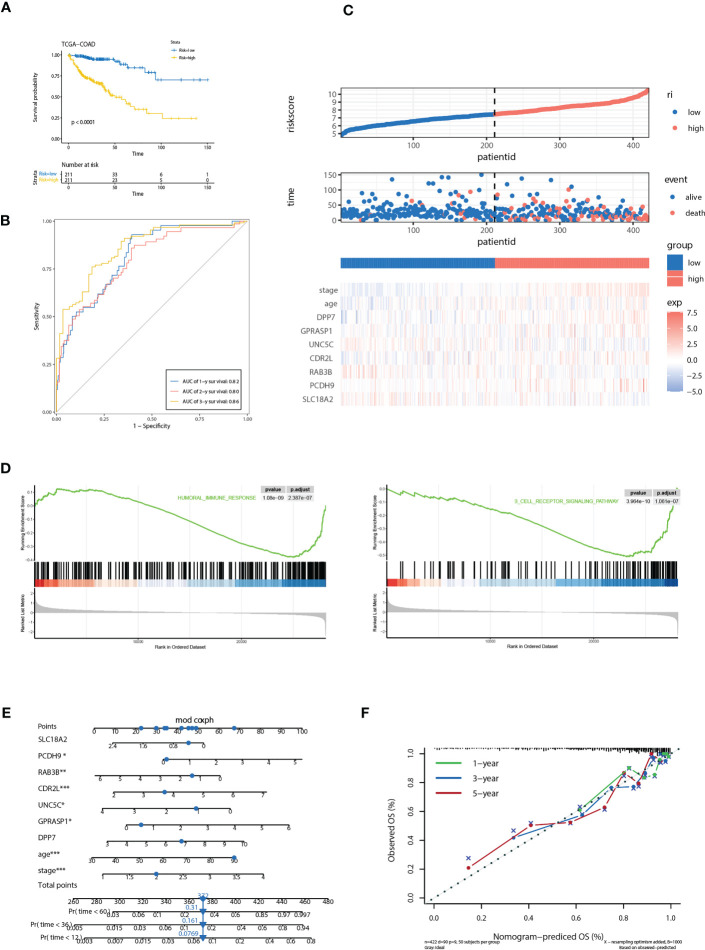
Correlation of the riskScore with clinicopathological features. **(A)** Kaplan-Meier plot of the riskScore signature in the TCGA cohort (log-rank test). **(B)** ROC curves for one-year, three-year and five-year overall survival prediction of the riskScore signature in the TCGA cohort. **(C)** Distribution of riskScore, survival status and the expression of prognostic CRGs. **(D)** Gene set enrichment analysis (GSEA) reveals two significantly activated signaling pathways including angiogenesis pathway and epithelial to mesenchymal transition pathway. **(E)** The nomogram to predict the probability of patient mortality using seven key molecules, age, and stage. **(F)** The calibration plot of nomograms between predicted and observed 1-year, 2-year, and 3-year outcomes. The 45-degree line represented the ideal prediction. *P<0.05, **P<0.01, ***P<0.001.

**Figure 6:**
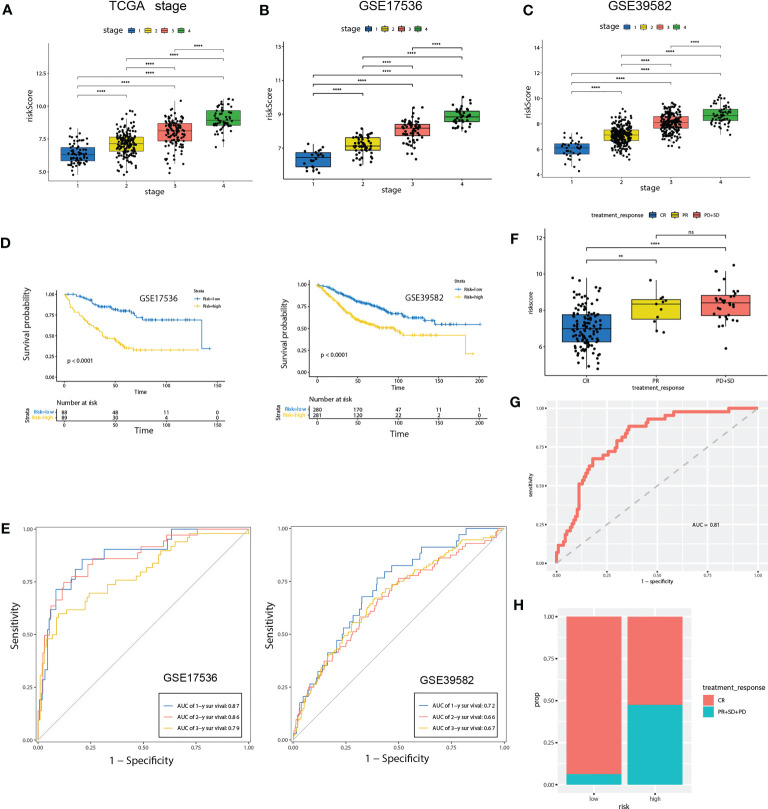
Validation of the prognostic prediction value of the riskScore in TCGA-COAD cohort, GSE17536 and GSE39582 dataset. **(A-C)** Differential levels of the riskScore between various TNM stages in TCGA-COAD cohort **(A)**, GSE17536 **(B)**and GSE39582 dataset **(C)**. **(D)** Kaplan-Meier plot of the riskScore signature in the GSE17536 and GSE39582 datasets (log-rank test). **(E)** ROC curves for one-year, three-year and five-year overall survival prediction of the riskScore signature in the GSE17536 and GSE 39582 datasets. **(F)** Differential levels of the riskScore between various treatment response in TCGA-COAD cohort. **(G)** ROC curves of the riskScore signature for complete remission prediction in the TCGA cohort. **(H)** The proportion of patients with response to chemical therapy in high or low riskScore groups. ns, not significant; **P<0.01, ****P<0.0001.

We further evaluated the predictive efficacy of riskScore for prognosis. Based on the model, cases in the training cohort were scored and divided into high- and low- risk group with the median riskScore as the cutoff. Survival analysis showed that cases with higher riskScore had a significant shorter OS compared with those with lower riskScore (*P*< 0.0001) (**Figure 5A**). ROC analysis revealed that AUC values of cases in the training cohort for 1-, 2-, and 3-year survival were 0.82, 0.80, 0.86 respectively, indicating good predictive efficacy of this model (**Figure 5B**). As the values of riskScore increased, mortality of those cases correspondingly increased (**Figure 5C**). Hierarchical clustering showed distinct expression patterns of seven key molecules between two groups (**Figure 5C**). Furthermore, levels of riskScore progressively increased with TNM stages of cases in the training cohort, which further consolidate its strong association with malignant phenotype (**Figure 6A**; **Supplementary Figure 3A**).

Gene set enrichment analysis (GSEA) reveals two immune related pathways, including humoral immune response and B cell receptor signaling pathway, were significantly attenuated, indicating that TME immune cells may be involved in high malignant phenotype of the high-risk cases (**Figure 5D**). To develop a clinically related quantitative method for predicting probability of patient mortality, we established the nomogram integrating all factors involved in the riskScore model (**Figure 5E**). The calibration plots demonstrated that the derived nomogram performed well compared to the ideal model (**Figure 5F**).

### Validation of the riskScore in two independent GEO datasets

To further confirm the prognosis predictive efficacy of the riskScore signature, we used GSE17536 and GSE39582 datasets as validation cohorts (n=177 and 561, respectively). Among different stages of COAD, cases with advanced TNM stages exhibited higher riskScore than in early TNM stages (**Figures 6B, C**). Higher riskScore was associated with advanced stages of T, N, M, stages in GSE39582 dataset, which is consistent with results in training cohort (**Supplementary Figure 3B**). Survival analysis showed OS of the high-risk group was significantly shorter than that of low-risk group (both *P*< 0.001) (**Figure 6D**). ROC analysis revealed that AUC value of the cases in the validation cohort for 1-, 2-, and 3-year survival were 0.87, 0.86, 0.79 for GSE17536, and 0.72, 0.66, 0.67 for GSE39582, respectively, indicating good predictive efficacy of the model (**Figure 6E**). We further evaluated association of riskScore with treatment response. Patients with complete remission (CR) exhibited a lower riskScore (**Figure 6F**). ROC analysis revealed that AUC value reached 0.81, indicating this model has excellent discriminant ability for CR (**Figure 6G**). CR rate was significantly enhanced in patients with low risk than those with high risk (93.75% vs 52.5%, **Figure 6H**).

### Evaluation of TME immune infiltration and checkpoints between the high- and low-risk groups

**Figure 7:**
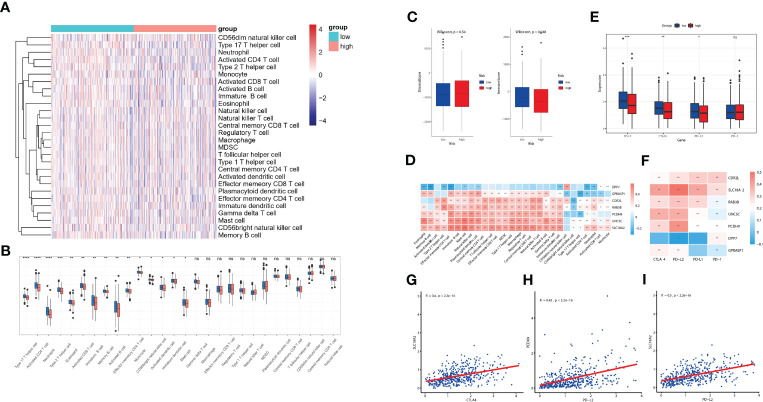
Evaluation of TME immune infiltration and checkpoints between the high- and low-risk groups. **(A)** Heatmap visualization of the relative abundance of 28 infiltrating immune cell types in the TCGA database data. Each small grid represents each immune cell, and the shade of color represents the infiltration level of this immune cell. The larger infiltration level is, the darker color will be (red is upregulated, and blue is downregulated). **(B)** Differences in 28 TME infiltration cells between high- and low-risk tissues (*P< 0.05; **P< 0.01; ***P< 0.001; **** *P*< 0.0001) **(C)** The difference of overall immune and stromal activity between high- and low-risk tissues using ESTIMATE algorithm. **(D)** The different expression levels of immune checkpoint molecules between high- and low-risk tissues **(E)** The correlation between each key molecule and each TME infiltration cell type. Red, positive; Blue, negative. **(F)** The correlation between the seven key molecules and immune checkpoint molecules. Red, positive; Blue, negative. **(G-I)** The correlations between SLC18A2 and CTLA4 **(G)**, PCDH9 and PD-L2 **(H)**, SLC18A2 and PD-L2 expression levels **(I)**. ns, not significant.

The diverse range of immune responses are largely attributed to the differential composition of immune cell population. It is uncertain whether those key molecules and riskScore signature are associated with TME immune infiltration, which may account for their association with prognosis. In this study, ssGSEA was used to determine the immune heterogeneity between riskScore-high group and -low group. We generated a heatmap to visualize the relative abundance of 28 infiltrating immune cells in each group. Of note, samples with low riskScore present with a high degree of immune cell infiltration, suggesting that they adopt an immune-hot phenotype, whereas those samples with high riskScore did the opposite (**Figure 7A**). We further evaluated proportion of immunoreactive and immunosuppressive cells in each of these populations. Consistent with the immune-hot phenotype of riskScore-low samples, the infiltrating cells were largely associated with immune activation (e.g., activated B cell, activated CD4 T cell, activated CD8 T cell, activated dendritic cell, etc.) (**Figure 7B**). An analysis based on the Tumor Immune Estimation Resource (TIMER; cistrome.shinyapps.io/timer) (16) further confirmed the relationship between seven key CRGs and the abundance of six immune cells (B cells, CD8+ T cells, CD4+ T cells, macrophages, neutrophils and dendritic cells) (**Supplementary Figure 4**). ESTIMATE algorithm analysis showed that immune scores, but not stromal scores, were significantly lower in riskScore-high group than in -low group (**Figure 7C**). We further used pearson correlation analysis to correlate key molecules with TME infiltrating cells, and multiple strong correlations were identified among them (**Figure 7D**). We also found that patients with lower riskScore is associated with elevated expression of PD-L1, CTLA4 and PD-L2, which is consistent with their immune-hot phenotype and suggested their potential vulnerability to Immune checkpoint inhibitors (ICI) treatments (**Figure 7E**). Expressions of seven key molecules were significantly correlated with immune checkpoint molecules (**Figure 7F**), of these, PD-1 and PD-L2 were consistently associated with seven key molecules. Correlations between SLC18A2 and PD-L2, PCDH9 and PD-L2, SLC18A2 and CTLA4 were particularly significant (**Figures 7G-I**).

### Validation of expression patterns of seven CRGs included in the riskScore

**Figure 8:**
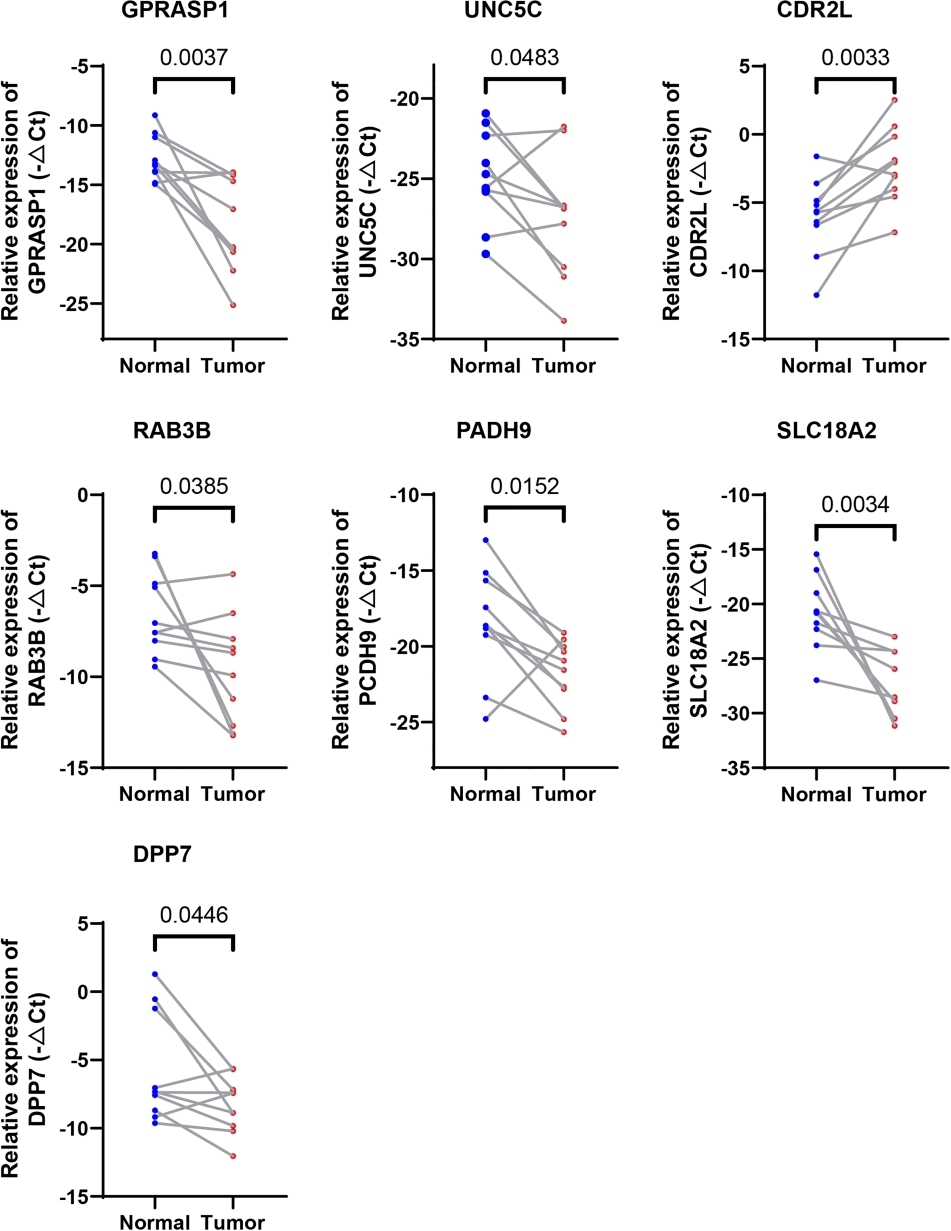
Validation of the expression levels of the seven cuproptosis-related genes in human tissues. Expression analysis of DPP7, GPRASP1, UNC5C, RAB3B, PCDH9, SLC18A2 and CDR2L were quantitated using qPCR in 10 pairs of cancer and paracancerous normal colon tissues.

We further evaluated the expressions of the seven cuproptosis-related genes involved in our riskScore signature. Ten paired cancer and paracancerous normal colon tissues were retrieved from COAD patients in our hospital. Consistent with results obtained from the TCGA-COAD cohort (**Supplementary Figure 1A**), six CRGs, including DPP7, GPRASP1, UNC5C, RAB3B, PCDH9, SLC18A2, were significantly downregulated, whereas CDR2L was upregulated in cancer tissues in comparison with paracancerous normal tissues (**Figure 8**). These results indicated that seven CRGs involved in our newly developed riskScore signature, may have regulatory effects in colon carcinogenesis, and may be explored as therapeutic targets against COAD.

### The seven CRGs involved in riskScore are regulated by copper

**Figure 9:**
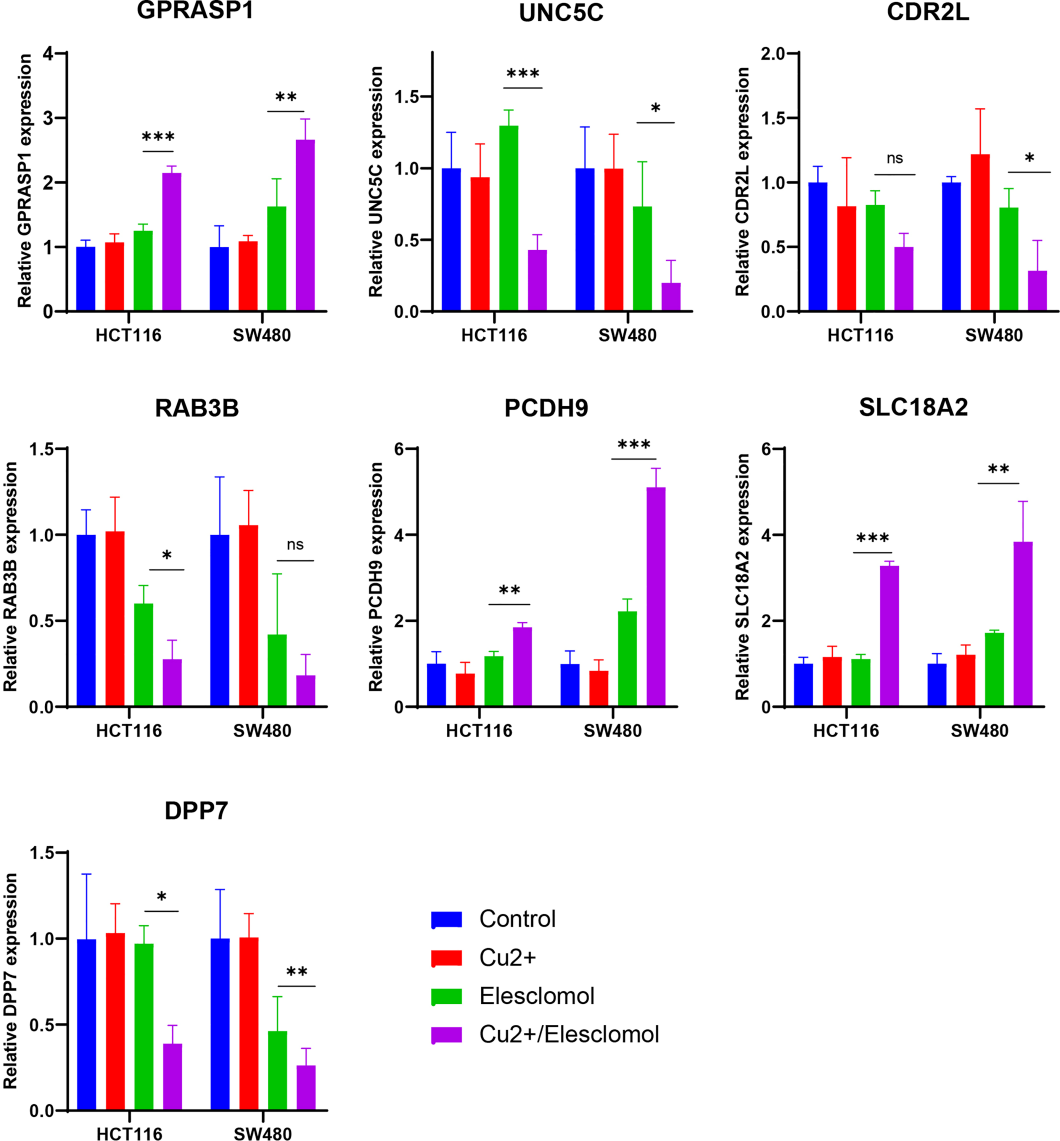
Regulation of cuproptosis on seven CRGs involved in riskScore. qPCR showed CRGs expression in HCT116 and SW480 cells treated with indicated drugs for 24 h (n=3). Drugs included CuCl_2_ (2mM), elesclomol (40 nM) alone or in combination. *P<0.05, **P<0.01, ***P<0.001. ns, not significant.

We next examined whether expressions of these 7 CRGs are altered in the setting of cuproptosis. Elesclomol is a copper ionophore that could shuttle copper into CRC cells and induce its overload, thus cuproptosis. We treated HCT116 and SW480 with copper chloride (2μM) in combination with different concentrations of elesclomol (10μM and 40μM) for 24 hours. RT-PCR analysis showed that expressions of all 7 CRGs were significantly altered (**Figure 9**). These results showed that these CRGs may be involved in the process of cuproptosis in COAD.

## Discussion

Cuproptosis is a newly discovered cell death form with emerging potential as a silver bullet in treatment against cancer. As apoptosis is the most common mechanism that mediates resistance to chemotherapy, identification of new cell death types, e.g., cuproptosis, will shed light on alternative treatment strategies for conquering drug resistance in refractory cancer cases.

Copper is an indispensable mineral nutrient for all organisms, homeostasis of which play critical roles in different physiological and pathological processes. Though previous publications established that copper overload was detrimental to cancer cells, a clear picture of the mechanisms underlying copper-induced toxicity has not yet emerged. Take CRC as an example, though numerous studies validated the anti-tumor effects of copper, distinct but contradictory death forms were suggested for underlying mechanisms, including ferroptosis (7, 17), autophagy (8), paraptosis (18), and etc. Recently, Tsvetkov et al. established a new death form named Cuproptosis, in which excess intracellular copper directly binds to lipoylated components of the tricarboxylic acid (TCA) Cycle. Ten genes, including FDX1, LIAS, LIPT1, DLD, DLAT, PDHA1, PDHB, MTF1, GLS and CDKN2A, were identified playing pivotal roles in cuproptosis.

A growing number of studies showed that prognostic models constructed based on RCD associated genes, including both coding and non-coding genes, may contribute greatly to the evaluation of patient prognosis, molecular characteristics and treatment modalities, and could be further translated into clinical applications (15, 19). Correspondingly, various prognostic models based on cuproptosis-regulated genes have recently been constructed in diverse tumors, including soft tissue sarcoma (20), melanoma (21), clear cell renal cell carcinoma (22), liver cancer (23), and so on. However, no research regarding the role that cuproptosis played in CRC has been reported. As studies regarding the field of cuproptosis are still in its infancy, those molecules constitute the complicated network are severely understudied. Our study first systematically investigates CRGs by calculating a coexpression correlation matrix in CRC, and established a riskScore model consisting of seven key CRGs and two clinical parameters (stage and age). Survival analysis suggested that patients with higher riskScore showed longer OS than those with lower riskScore. ROC analysis revealed that AUC values of cases in the training cohort for 1-, 2-, and 3-year survival were 0.82, 0.80, 0.86 respectively, indicating the good predictive efficacy of this model. Correlations with clinical features showed that higher riskScore was significantly associated with advanced stages of T, N, M, and TNM stages. Consistent results were further validated in two independent GEO datasets. Regarding associations with treatment response, CR rate was significantly enhanced in patients with low risk than those with high risk. Through integrating those factors involved in riskScore, we established a quantitative nomogram, which improved the performance and facilitated clinical use of the riskScore.

Numerous studies revealed the essential roles of RCDs in innate immunity and antitumor effects (15, 19); however, few studies reported the potential role of CRGs in immunotherapy, with none regarding CRC. Several lines of evidence support the potential involvement of our riskScore model in CRC TME immunity. First, cases in the TCGA cohort were scored and divided into high- and low- risk group with the median riskScore as the cutoff, and Gene set enrichment analysis (ssGSEA) showed that high-risk group presents with an immune-cold phenotype. Second, Gene set enrichment analysis (GSEA) reveals that certain immune-related pathways were significantly associated with riskScore levels. Third, ESTIMATE algorithm analysis showed that immune scores varied widely in different riskScore groups. Therefore, we quantified the proportions of tumor infiltrating immune cells in the low- and high-risk groups to evaluate their associations. Emerging evidence supported the potential role of B cells in cancer immune response (24, 25). In soft-tissue sarcomas, B cell infiltration was an independent and the strongest prognostic factor for good prognosis and correlated with improved response to PD-1 blockade (26). Consistently, favorable prognosis was also identified in CRC patients with enhanced tumor-infiltrating B cells (27), which is the case in metastatic CRC, that increased B cells infiltration was associated with lower risk of recurrence and improved survival (28). These data established B cells as a novel target for immunotherapy and could be a strong weapon against cancer. In our study, patients with lower riskScore, showed higher infiltration of B cells, suggesting that they play an anti-tumor role in CRC development. Cytotoxic T cells which corresponds to our finding of more activated CD8+ T cells in patients with lower riskScore compared with those in the high-risk group. ICIs based immunotherapy has provided a new direction for tumor treatment in various cancer types (29, 30), including CRC (31). In the present study, higher levels of ICIs, including PDL1, CTLA4, and PDL2, were observed in patients with lower riskScore group, indicating their potential good response to immune checkpoint blockade.

Recently, several papers have been published on the use of a model based on cuproptosis-associated genes for predicting prognosis (32–35). However, these papers suffer from several limitations, including a small number of genes in their signature, relatively low prognostic power, and illogical combination with other cell death types like ferroptosis. Additionally, no experiments on colorectal cancer cells have been conducted regarding CRGs. Our work is unique in that we evaluate the association between our predictive score and treatment response. Our results show excellent discriminant ability for complete remission, providing valuable information to clinicians for therapeutic selection.

There are several strengths in our study. First, our study is the first to systematically investigate CRGs by calculating a coexpression correlation matrix in CRC. A riskScore model was constructed with good predictive efficacy for survival and TME immune infiltration. Second, clinical significance of our model was validated in two independent GEO datasets, indicating stability and universality of our model. Third, we established a nomogram involving all factors of this model, which further improved clinical applicability of the riskScore. Last but not least, we examined expressions of these CRGs and validated that their levels were significantly altered in cancer tissues in comparison with paracancerous normal tissues. *In vitro* studies also showed that these CRGs may be altered in the setting of copper overload. All these findings expand our knowledge regarding the potential roles of seven key CRGs in the process of cuproptosis. Certainly, there are some limitations of current study. All analysis were conducted based on data extracted from retrospective public databases with possible selection bias, large-scale prospective studies are needed to confirm our results.

In conclusion, this study is the first that constructed a riskScore based on CRGs that exhibited good predictive performance for OS in CRC. Future studies are warranted to dissect the underlying mechanisms by which these CRGs get involved in the process of cuproptosis and explore their potential roles as therapeutic targets against CRC. We hope that this riskScore can be validated and used in future clinic to guide therapeutics selection.

## Data availability statement

The datasets presented in this study can be found in online repositories. The names of the repository/repositories and accession number(s) can be found in the article/**Supplementary Material**.

## Ethics statement

The studies involving human participants were reviewed and approved by Shanghai Changhai Hospital Ethics Committee. The patients/participants provided their written informed consent to participate in this study.

## Author contributions

This research was conducted in collaboration with all authors. LL, FS and FK performed the data curation and analysis. YS and YF analyzed and interpreted the results. YD and FL drafted the manuscript. XK finally reviewed the manuscript. All authors contributed to the article and approved the submitted version.

## Supplementary material

Supplementary Figure 1 The expression level and survival of 7 pivotal CRGs. **(A)** Seven out of ten reported CRGs were differentially expressed between tumor and paired normal cases (*P*<0.05). **(B)** Kaplan-Meier plot of seven genes in TCGA-COAD (log-rank test).

Supplementary Figure 2 The expression level and survival of seven prognosis-related CRGs. **(A)** Seven prognosis-related CRGs were differentially expressed between tumor and paired normal cases (*P*<0.05). **(B)** Kaplan-Meier plot of seven prognosis-related CRGs in TCGA-COAD (log-rank test).

Supplementary Figure 3 The relationship between the riskScore and TNM stage. **(A)** Differential levels of the riskScore between various T, N, M stage in TCGA-COAD cohort. **(B)** Differential levels of the riskScore between various T, N, M stage in GSE39582 dataset.

Supplementary Figure 4
**(A-G)** Correlation between each key molecule expression and immune infiltration in TCGA-COAD in the TIMER database.
