# Supplementary figures and images for: Characterization of a cuproptosis-related signature to evaluate immune features and predict prognosis in colorectal cancer
Source: Front Oncol. 2023 Jun 13;13:1083956. doi: 10.3389/fonc.2023.1083956 (PMC10299831; doi:10.3389/fonc.2023.1083956)

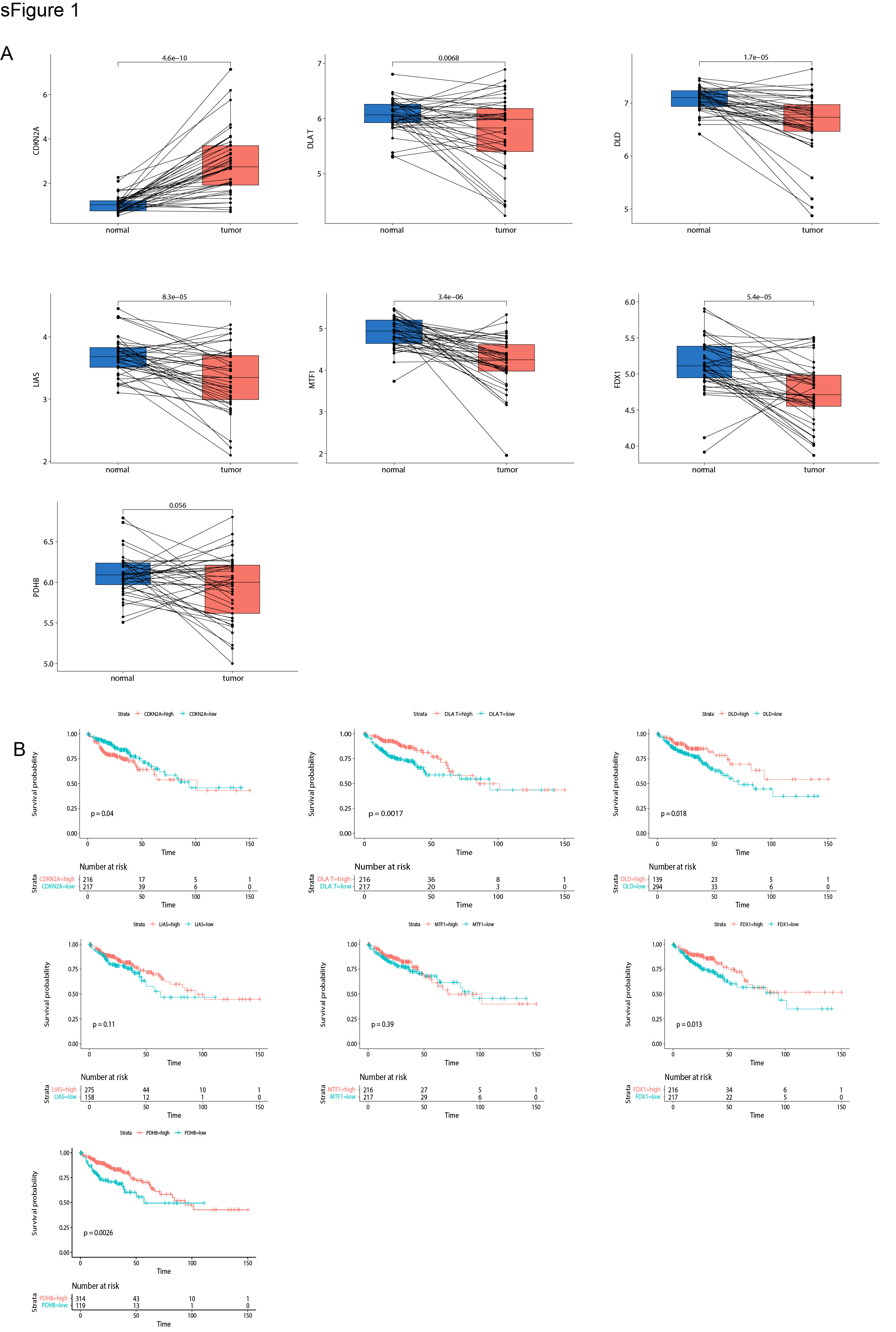

Supplement: Supplementary Figure 1 — The expression level and survival of 7 pivotal CRGs. (A) Seven out of ten reported CRGs were differentially expressed between tumor and paired normal cases (P<0.05). (B) Kaplan-Meier plot of seven genes in TCGA-COAD (log-rank test). [file Image_1.jpeg]

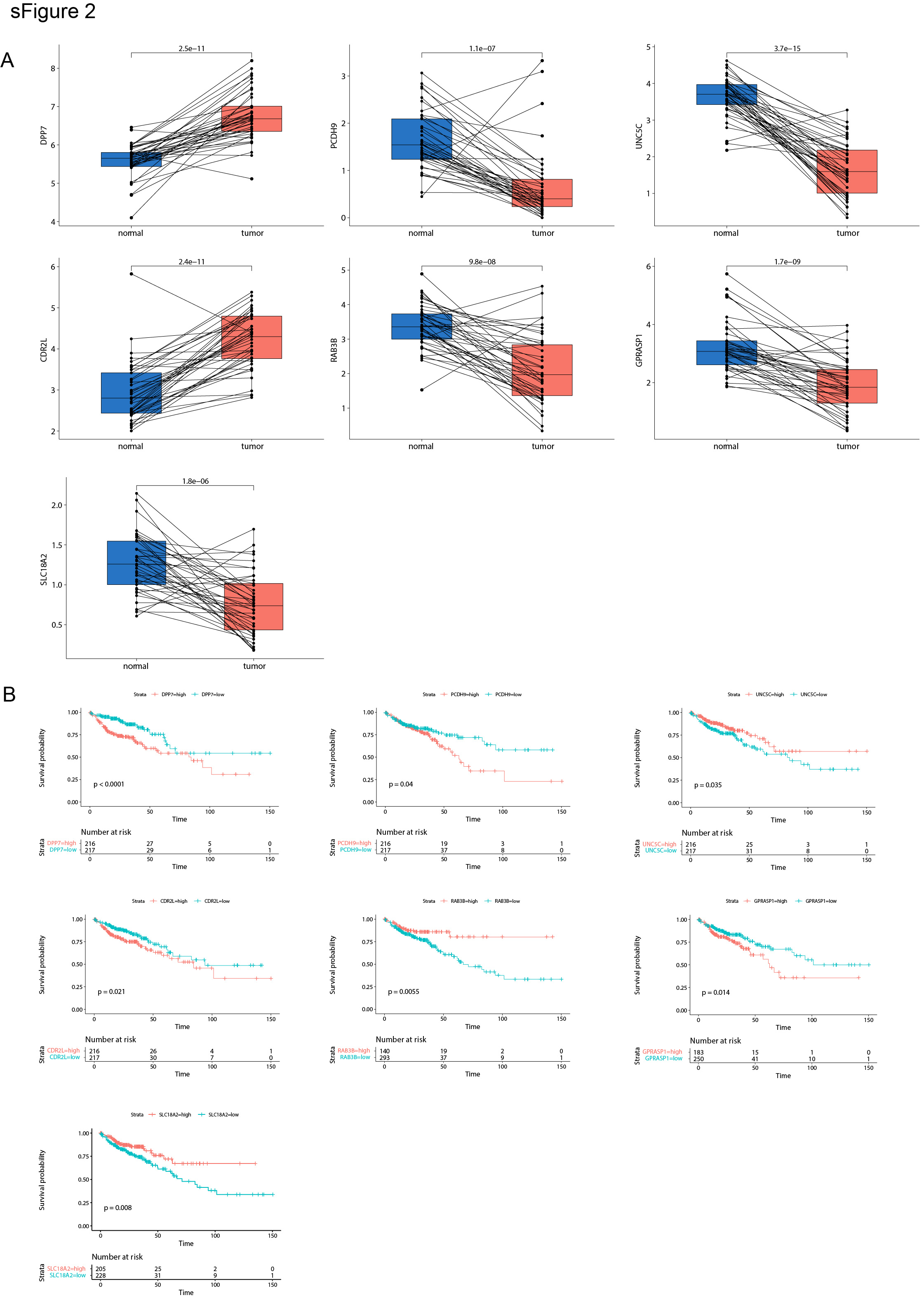

Supplement: Supplementary Figure 2 — The expression level and survival of seven prognosis-related CRGs. (A) Seven prognosis-related CRGs were differentially expressed between tumor and paired normal cases (P<0.05). (B) Kaplan-Meier plot of seven prognosis-related CRGs in TCGA-COAD (log-rank test). [file Image_2.jpeg]

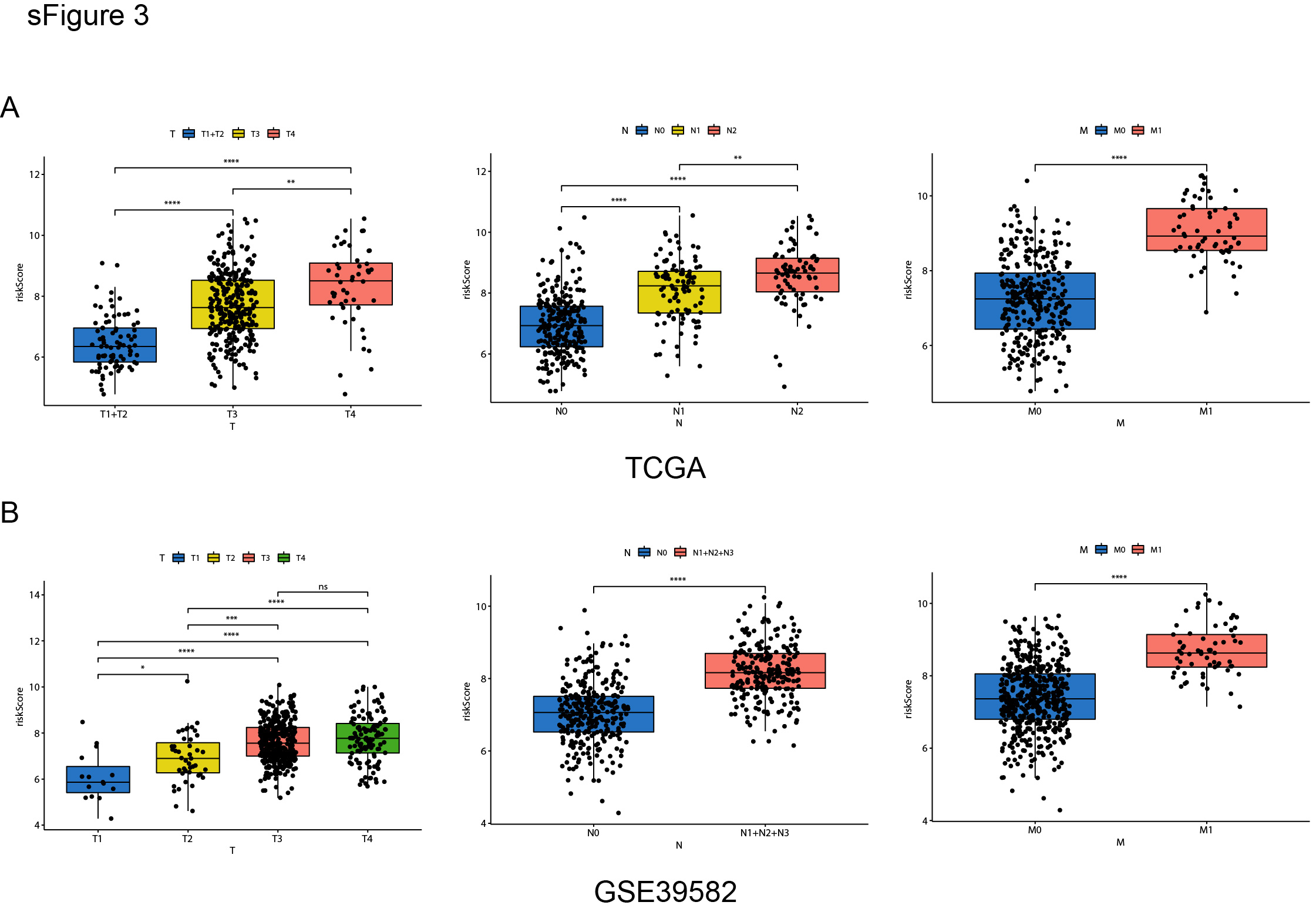

Supplement: Supplementary Figure 3 — The relationship between the riskScore and TNM stage. (A) Differential levels of the riskScore between various T, N, M stage in TCGA-COAD cohort. (B) Differential levels of the riskScore between various T, N, M stage in GSE39582 dataset. [file Image_3.jpeg]

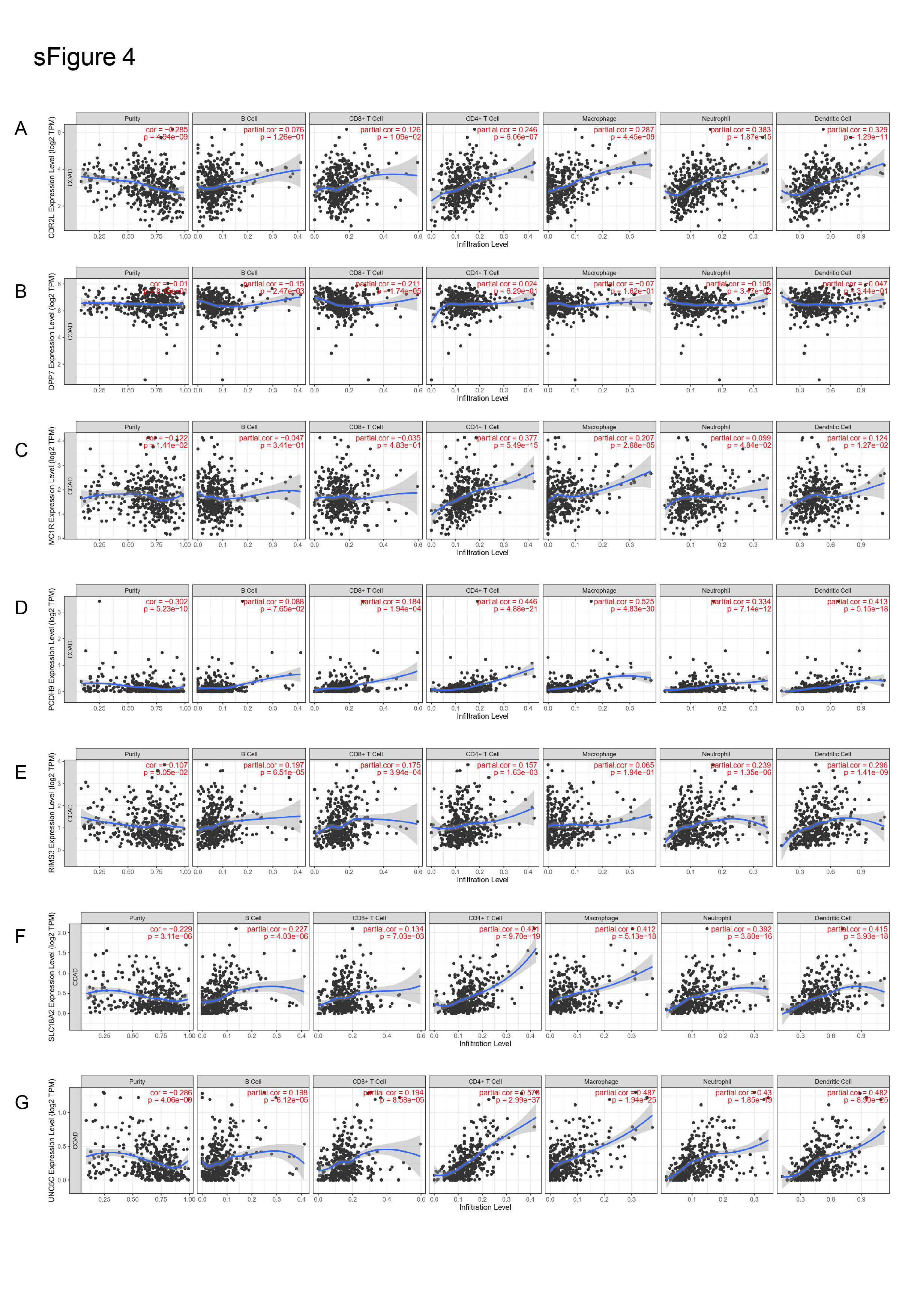

Supplement: Supplementary Figure 4 — (A-G) Correlation between each key molecule expression and immune infiltration in TCGA-COAD in the TIMER database. [file Image_4.jpeg]
